# Problematizing Digital Research Evaluation using DOIs in Practice-Based Arts, Humanities and Social Science Research

**DOI:** 10.12688/f1000research.6506.1

**Published:** 2015-07-07

**Authors:** Muriel Swijghuisen Reigersberg

**Affiliations:** 1 Research Office, Goldsmiths', University of London, London, SE14 6NW, UK

**Keywords:** Open Access, Digital Identifiers, Arts, Humanities, Social Science

## Abstract

This paper explores emerging practices in research data management in the arts, humanities and social sciences (AHSS). It will do so vis-à-vis current citation conventions and impact measurement for research in AHSS. Case study findings on research data inventoried at Goldsmiths’, University of London will be presented. Goldsmiths is a UK research-intensive higher education institution which specialises in arts, humanities and social science research. The paper’s aim is to raise awareness of the subject-specific needs of AHSS scholars to help inform the design of future digital tools for impact analysis in AHSS.

Firstly, I shall explore the definition of research data and how it is currently understood by AHSS researchers. I will show why many researchers choose not to engage with digital dissemination techniques and ORCID. This discussion must necessarily include the idea that practice-based and applied AHSS research are processes which are not easily captured in numerical ‘sets’ and cannot be labelled electronically without giving careful consideration to what a group or data item ‘represents’ as part of the academic enquiry, and therefore how it should be cited and analysed as part of any impact assessment.

Then, the paper will explore: the role of the monograph and arts catalogue in AHSS scholarship; how citation practices and digital impact measurement in AHSS currently operate in relation to authorship and how digital identifiers may hypothetically impact on metrics, intellectual property (IP), copyright and research integrity issues in AHSS.

I will also show that, if we are to be truly interdisciplinary, as research funders and strategic thinkers say we should, it is necessary to revise the way we think about digital research dissemination. This will involve breaking down the boundaries between AHSS and other types of research.

## Introduction

This paper explores emerging practices in research data management in the arts, humanities and social sciences (AHSS). It will do so vis-à-vis current citation conventions and impact measurement for research in AHSS. Case study findings on research data inventoried at Goldsmiths’, University of London will be presented. Goldsmiths is a UK research-intensive higher education institution which specialises in arts, humanities and social science research.

The subject of this paper is a topical one in the UK, where research in Universities is publically funded. Government and research council funders are asking that Universities in receipt of research income demonstrate how their funding is used to generate new knowledge and positive impact in all disciplines, including the arts, humanities and social sciences. The impact that this new knowledge creation and its dissemination are having must be recorded and where possible, quantified. This quantitative information can thereafter be used to help inform future research strategies on a variety of levels. It might also be used, some tentatively suggest, to complement peer review in future research excellence frameworks.

Some of this quantifiable information for research assessment might be delivered through various types of metrics, based on digital information made openly available. This could include bibliometrics; a quantitative analysis of research literature and citation rates or altmetrics, which incorporates for example social media analyses and download rates of visual research-related materials, alongside citation rates.

This way of measuring research impact and excellence however, is not yet as refined as some researchers might like it to be. Firstly, the data on which metrics relies must be available in a digital format and easily accessible. Secondly, data must be unambiguously linked to its creator(s) through unique researcher identifier numbers, such as those provided by ORCID. Some argue metrics works better for those disciplines that have focussed more heavily on digital dissemination strategies and open access publication methods. It is argued that some forms of research, such as practice-based research, do not naturally lend themselves well to digital capturing for impact measuring purposes. The upshot of this is that those disciplines that are less digitally oriented, are likely to obtain unhelpful metric ratings. This in turn it is feared, will lead to reductions in public funding, if metrics were to be used to allocate financial resources in future. Some even suggest that this then in turn, might jeopardise the diversity of UK research, diminishing arts and practice-based research activity and jeopardising the sustainability of smaller specialist higher education institutions. As a result, the Higher Education Funding Council for England (Hefce) conducted two independent reviews on the suitability of metrics for research and impact assessment purposes: one between 2008 – 2009, and another soon to be published in July 2015.

Disciplinary languages also determine whether or not AHSS researchers are likely to engage with metrics. Many AHSS researchers do not define their research outputs as ‘data’. Neither do many communicate their research enquiries in writing, making bibliometrics problematic. Often, when they do write, AHSS researchers publish monographs or book chapters. Monographs as yet are not widely available in an open access format, again impacting on the ability of the research therein to be captured by digital tools. Lastly, questions of authorship, copyright and ownership arise. Often AHSS research is co-created with the help of non-researchers as well as co-investigators. This impacts on individual incomes and research integrity where the sharing and citation of data is concerned. It may also impinge on research data management strategies for interdisciplinary projects where multiple outputs of different kinds are produced.

It is the above issues that I will explore in this paper. I will argue that if we are to be truly interdisciplinary, it is necessary to revise the way we think about digital research dissemination and how we write and talk about it. This will involve breaking down the boundaries between AHSS and other types of research.

Although presented here as an opinion piece, it is not so much an opinion as a record of the state of play with regards to emerging practices, theory and ideas on digital publication, ORCID numbers and digital object identifiers and how these might complement other mechanisms which enhance digital research impact and discoverability of AHSS research. This article does not pretend to offer a complete view of all emerging practices across AHSS disciplines in the UK. Neither does it suggest that digital citation and discoverability are the only ways in which impact can be achieved. Instead it will give an overview of some of the specific debates pertaining to digital dissemination that researchers and administrators are having at Goldsmiths, University of London and how staff are engaging with open access, open data, digital object identifiers (DOIs) and ORCID numbers.

### ORCID, Definitions of Data in AHSS Research

At Goldsmiths, University of London, not many researchers have created their individualised ORCID numbers yet and neither do they, by and large, label their data with digital object identifiers (DOIs), apart from possibly their journal articles, which of course contain processed data. This is because many researchers active in arts, humanities and social science (AHSS) research do not identify their research outputs (e.g. field notes, monographs, art work sketches, film rushes, interview materials etc.) as research data. The term ‘data’, to them has a much narrower definition, belonging only to the professional jargon used by researchers working in (bio) medical and other scientific fields (e.g. quantitative, numerical data sets, graphs and pie charts). Other AHSS researchers see ‘data’ as only being synonymous with personal data: personal information about research participants, such as names and dates of birth, subject to safeguarding by the data protection act.

The definition of what might be classified as research data, however, could be much broader if conceived of creatively. The first mission of any digital advocate therefore, is to clarify with the help of researchers themselves, what might count as research data in their disciplines and how this could be labelled appropriately, digitised, archived and maintained as necessary, to document research processes and methods. This process of defining and scoping research data must meet the needs of project-specific research enquiries without letting administrative requirements take the upper hand or imposing non-AHSS data collection models where this is not appropriate.

To some extent the question of what counts as data in visual arts research was already addressed at Goldsmiths. Between 2011–2013, Goldsmiths participated in a Joint Information Systems Committee (JISC) funded project called KAPTUR together with Glasgow School of Art, University for the Creative Arts and University of the Arts London. KAPTUR built on the work undertaken by the Digital Curation Centre and was led by the Visual Arts Data Service. The KAPTUR project sought to: “investigate the current state of the management of research data in the arts; develop a model of best practice applicable to both specialist arts institutions and arts departments in multidisciplinary institutions; and to apply, test and embed the model with four institutional partners ( http://www.vads.ac.uk/kaptur/about.html)”. The project helped question definitions of data and provide examples of what might count as data and how it might be managed.

KAPTUR’s findings, useful toolkits, reports and outcomes have not filtered through to most visual arts researchers however and few therefore have embraced the idea that the definition of research data can be broadened. Those that have, however: are struggling with IP and copyright issues for digitised work; have no time to digitise analogue research outputs to be able to create digital object identifiers; or may not publish in the digital domain, meaning that digital identification and metrics cannot currently be used effectively to assess any potential impact being created.

Consequently, many AHSS researchers feel ORCID numbers and digital object identifiers are simply not relevant to what they currently do. As much AHSS research remains unfunded there are also no contractual or funder obligations to which researchers must adhere which stipulate that research data must be open access or discoverable. This reluctance to engage with digital dissemination and citation practices is especially in evidence when researchers work in disciplines that still rely on the production of practice-based outputs and the publication of monographs as the most important esteem indicators and research outputs in their fields.

### Monographs in AHSS Research

Goldsmiths’ researchers publish many monographs. Monographs contain research data: images of art works; creative writing outputs; and (auto) biographical details for example. The author is not always the copyright owner of this data or indeed its creator, and will often have to gain permission to use information for the purposes of publishing their monograph. What is owned by the author is the intellectual theory and often text.

Academic monographs are still predominantly issued in non-open access formats. If published in the digital domain, proprietary formats and specific software are used. These are unlikely to promote sustainability of digital research data in the open access domain. The challenge is compounded by the current lack of digital research data for monographs that might easily be labelled and referenced electronically. Here it is worth referring to the January 2015 Crossick report on open access monographs generated by the Higher Education Funding Council England (Hefce).

Whilst recognising the importance of monographs to AHSS scholars, the Hefce report identified the limitations of hard copy formats. Those relevant to us here include: a) the fact that video, audio and other examples cannot be embedded in hard-copy monographs; b) text-mining options and easy ways of measuring citation levels and impact rates are absent; c) the fact that hard copy monographs are not ‘living’ documents. Comments and reviews cannot be easily shared, updates require new editions and comparisons of passages and ideas are not quickly communicated. It is these limitations of the monograph that researchers at Goldsmiths are wishing to explore in collaboration with publishers, computing and legal experts.

The challenges identified by the Hefce report with regards to labelling research data and making monographs open access are several. Those highlighted by the report are, for example, that academically authored exhibition catalogues are part of business models for Independent Research Organisations (IROs) and galleries. Making exhibition catalogues and the research data openly accessible will reduce the vital income received by IROs such as the Tate Galleries. Additionally, data in catalogues and creative writing outputs have often been generated by people other than the catalogue author. This raises copyright, intellectual property and revenue challenges impacting on the licensing and sub-licensing of research data such as images and musical examples if researchers wanted to include certain materials in their open access monograph using digital object identifiers. Careful consideration must therefore be given to labelling research data before joining it to researcher ORCID numbers and making it open access if in monograph or catalogue format.

### The analogue, licencing, authorship and ethics

Questions of research ethics, integrity and licencing also come in to play when labelling practices are considered. Pictures, images and text may constitute to a representation of something or someone. Not the actual object or person. Practice-based researchers often argue that a (digital) image or recording of their analogue, and possibly temporaneous art work is a different object epistemologically to the actual, physical work and therefore cannot be labelled as being the same item. Similarly, where open access is an option, anthropologists and ethnographers frequently opt for non-derivative licences meaning no materials based data/text can be created derived from the original text. This is important where non-academic research collaborators agree to participate on the basis that they are represented fairly and where these collaborators often have a say in how their interview excerpts, musical materials and images (that is to say, research data collected by the researcher) are used and placed in texts. Re-using research data uncritically, or labelling it with object identifiers often runs contrary to the highly personalised material that is being explored, which belongs to both the researcher and his/her participants at the very least and in some cases to the research participant alone, who is sharing it with the researcher in good faith. AHSS authors therefore choose the most restrictive licensing options in order to do no harm. This ethical priority reduces the re-use and therefore citation options available for their data and as a result the potential for metrical impact.

Questions of authorship and citation surface as well. In the sciences definitions of what constitutes ‘authorship’ vary across disciplines and between journals. Citation practices are also not standardised. Guidelines do exist, however. By way of contrast, in AHSS research very few, if any, definitions and guidelines of what constitutes to authorship exist. If one were to apply certain biomedical models of authorship definition to AHSS research, many non-academic research participants would technically qualify as co-authors of research papers as they helped shape research data via their active, sometimes non-anonymous, participation in the research enquiry, particularly in applied, bottom-up, process-oriented research enquiries.

Whilst acknowledging research participants’ input and possible co-authorship of research papers and data may be a more accurate and ethical reflection of their role in the research, it could also potentially create logistical challenges in the domains of IP, copyright, ethics and citation. Ethical considerations need to inform citation and author definition practices. Whilst it might seem like a good idea to measure impact through non-academic authorship, citation and engagement in the way hinted at above, this approach should not be recommended without careful ethical screening addressing questions of anonymity and equitable data sharing and ownership that are likely to arise, amongst many other hurdles.

### Metrics, impact and digital dissemination in AHSS

Another challenge to be overcome is that of the use of metrics in assessing research quality and impact. ORCIDs and DOIs will be especially helpful in collecting statistical information quickly and digitally on how often research is being cited, and accessed and may go some way, so the argument goes, to showing how much impact is being achieved. Metrics however, is only one way in which impact becomes measurable and for AHSS researchers, it is thought to be misleading and ineffective. In a response to Hefce’s consultation on the use of metrics in assessment of research quality and impact, many Goldsmiths staff remained unconvinced that metrics could be used to conclusively prove research excellence or impact. Implicitly therefore, they had little faith in the use of DOIs and ORCID numbers as a way of improving impact analyses through metrics, although some did agree it would improve the visibility of research outputs and data. Most felt though that discoverability should not be equated with quality or impact *per se*.

Metrics such as citation rates and journal impact factors, they observed, operate differently not only according to discipline (e.g., between psychology and literature), but also differ significantly between varying branches of the same discipline. Psychology, for example, is arguably a more diverse discipline than some, so indices like citation factors need to be interpreted very carefully even within different sub-disciplines to allow for meaningful 'like for like' comparisons (e.g., neuroscience journals typically have much higher impact factors than social psychology journals).

The Media, Communication and Cultural Studies Association’s (MeCCSA) REF consultation, compiled by Prof Golding and reiterated in the Goldsmiths’ response to the Hefce metrics consultation by MeCCSA members employed at Goldsmiths, yielded various anonymised comments from researchers. It was pointed out that evidence “suggests that variations in citation practices occur within disciplines as much as across disciplines, so the issue of calibration cannot simply be to the average for the subject as is proposed in science subjects. Staff felt it difficult to envisage a reliable way in which to develop disciplinary citation norms in interdisciplinary areas against which to compare individual counts. This would be especially true in fields such as media and communications which encourage publication across a very wide range of outlets in the arts, humanities, and social sciences. The usual suggestion of Web of Science (Thomson Scientific) as the database to be used for calibration of citation counts is acknowledged to be problematic. Web of Science is demonstrably incomplete in many areas ( http://www.meccsa.org.uk/pdfs/REF-Consultation.pdf)”. Colleagues were unconvinced any database was complete and therefore figures not necessarily accurate or useful.

Others Goldsmiths’ colleagues felt that in AHSS research the number of citations was not a conclusive indicator of research merit, value or impact. A colleague commented that in the humanities (literary studies in particular), no publication is ever really superseded or made obsolete, nor is the author-critic irrelevant to the argument; the argument is very often his/her interpretation and appreciation of certain phenomena; in the sciences the assumption is that scientists report hard facts/results of experiments, not their idiosyncratic and poetic take on the set of data. Once a set of data or theory is superseded, scientific research tends no longer be cited or becomes part of what ‘everybody already knows’ (see Latour & Woolgar, 1986). This is not true for a lot of AHSS research and so the citation of data or the linking of outputs with ORCIDs might be of limited use it was felt.

The intellectual debates surrounding digital dissemination that are flourishing at Goldsmiths will inform practical digital dissemination strategies that the University as a whole will adopt in future. Intellectually these same debates seek to influence emerging theory in digital scholarship and dissemination in AHSS subjects more generally.

### Interdisciplinarity, impact, Goldsmiths and digital dissemination

Despite this scepticism and caution, new developments have begun to flourish. During recent data management scoping exercise, it became apparent that Goldsmiths researchers are engaging with digital dissemination practices quite effectively. This is especially the case where researchers are working on projects that are interdisciplinary, well-funded and usually include an element of non-AHSS research.

For example, Goldsmiths hosts a large Arts and Humanities, Research Council (AHRC) grant. Its research team actively seek to use and create scientific computing tools to collect and analyse large amounts of data to help further musicological analyses and practice. One such undertaking is the ‘ *Transforming Musicology’* project ( Box 1).

Box 1. **Excerpt from the ‘Transforming Musicology’ website ( http://www.transforming-musicology.org/about/) – Principal investigator: Professor Tim Crawford**

This research project explores how software tools developed by the music information retrieval (MIR) community can be applied in musical study. Specifically the project seeks to:

- enhance the use of digitally encoded sources in studying 16th-century lute and vocal music and using such sources to develop new musical pattern matching techniques to improve existing MIR tools;
- augment traditional study of Richard Wagner’s leitmotif technique through audio pattern matching and supporting psychological testing;
- explore how musical communities on the Web engage with their music by employing MIR tools in developing a social platform for furthering musical discussion online.

A key technological contribution of *Transforming Musicology* is the enhancement of Semantic Web provisions for musical study. This involves augmenting existing controlled vocabularies (known as ontologies) for musical concepts, and especially developing such vocabularies for musical discourse (both academic and non-academic). It will also involve developing and promoting methods to improve the quality and accessibility of music data on the Web; especially the accessibility for automatic applications, following techniques known as linked data.

The project relies heavily on the digital labelling of musical units to help catalogue and identify compositional structures and musical pieces. The research has the potential to inform debates on musical performance, copyright, composition and musical analysis amongst other areas. To this end it will develop open source software tools as well. The process of identifying and labelling musical units with URIs means that this project has a large number of data sets and individual data items which might potentially be cited and accessible in audio format in the planned monograph for this large grant. The grant’s research team are presently considering the possibility of engaging with publishers to explore open access monograph formats so that they might include digital data sets. The creation of digital data sets and an open access monograph, in turn, provide a significant impetus for the research team to consider adopting ORCID numbers so that URIs and DOIs might be linked to their names and the grant. If data sets and digital tools were made available they would also benefit non-academic and amateur music groups, such as the lute-players with which the principal researcher works. For open access sharing to be made a reality however, careful consideration will need to be given to how research data sets are shared in the monograph and whether or not this might contravene existing copyright legislation, for example, as data is compiled from existing musical pieces. Until adequate sharing mechanisms are explored, it may not be possible to freely share the data accumulated during the project’s lifespan.

Another initiative taken by Goldsmiths’ researcher Joanna Zylinska and her team (Professor Joanna Zylinska, Dr Kamila Kuc, Jonathan Shaw, Ross Varney, Dr Michael Wamposzyc. Project advisor: Professor Gary Hall), includes the creation of an open book, *Photomediations*. The project redesigns a coffee-table book as an online experience to produce a creative resource that explores the dynamic relationship between photography and other media. *Photomediations: An Open Book* uses open (libre) content, drawn from various online repositories (Europeana, Wikipedia Commons, Flickr Commons) and tagged with the CC-BY licence and other open licences. In this way, the book showcases the possibility of the creative reuse of image-based digital resources.

Through a comprehensive introduction and four specially commissioned chapters on light, movement, hybridity and networks that include over 200 images, *Photomediations: An Open Book* tells a unique story about the relationship between photography and other media. The book’s four main chapters are followed by three ‘open’ chapters, which will be populated with further content over the next 18 months. The three open chapters are made up of a social space, an online exhibition and an open reader. A version of the reader, featuring academic and curatorial texts on photomediations, will be published in a stand-alone book form later in 2015, in collaboration with Open Humanities Press.

*Photomediations: An Open Book’s* online form allows for easy sharing of its content with educators, students, publishers, museums and galleries, as well as any other interested parties. Promoting the socially significant issues of ‘open access’, ‘open scholarship’ and ‘open education’, the project also explores a low-cost hybrid publishing model as an alternative to the increasingly questioned traditional publishing structures. Photomediations: An Open Book is a collaboration between academics from Goldsmiths, University of London, and Coventry University. It is part of Europeana Space, a project funded by the European Union's ICT Policy Support Programme under GA n° 621037. It is also a sister project to the curated online site Photomediations Machine: http://photomediationsmachine.net. This example provides a good model of how AHSS researchers in the visual arts might approach the production of open access monographs and arts catalogues, where licencing and copy right issues are very much foregrounded.

A third example of Goldsmiths engagement with open access dissemination and data is that of work led by Dr Jennifer Gabrys and her team on an ERC funded project called *Citizen Sense* in the Sociology department ( Box 2).

Box 2. **Excerpt from the ‘Citizen Sense’ website ( http://www.citizensense.net/sensors/environmental-data/). Leader: Dr Jennifer Gabrys**

The project, which runs from 2013–2017, investigates the relationship between technologies and practices of environmental sensing and citizen engagement. Wireless sensors, which are an increasing part of digital communication infrastructures, are commonly deployed for environmental monitoring within scientific study. Practices of monitoring and sensing environments have migrated to a number of everyday participatory applications, where users of smart phones and networked devices are able to engage with similar modes of environmental observation and data collection. Such “citizen sensing” projects intend to democratize the collection and use of environmental sensor data in order to facilitate expanded citizen engagement in environmental issues.

The team examine how effective citizen sensing practices are in not just providing “crowd-sourced” data sets, but also in giving rise to new modes of environmental awareness and practice. Through intensive fieldwork, study and use of sensing applications, the project areas set out to contextualize, question and expand upon the understandings and possibilities of democratized environmental action through citizen sensing practices.

As part of their studies the research team on *Citizen Sense* collect live scientific data on for example air quality, using sensor devices. The team has now developed a website which visualises air quality data so that the general public can view the crowd sourced results. The interdisciplinary scope of this large project therefore means that the research data generated comes in a format that is more akin to data generated in science environments as opposed to AHSS disciplines. Therefore the collection, labelling, storage and archiving of this data using DOIs and attaching these to ORCIDs might therefore usefully draw on practices established in non-AHSS domains. This in turn could potentially enhance the visibility and impact of this research both environmentally and academically.

## Conclusion

Whilst many AHSS researchers at Goldsmiths remain sceptical about the use of ORCID numbers and digital object identifiers to enhance impact, the Goldsmiths examples show that there are distinct possibilities for their ability to enhance the visibility of on-line research outputs such as open access monographs, digital musical data and sociologically inspired scientific data. These examples, however, are sourced from projects that are highly interdisciplinary and well-funded, drawing on collaborations and resources not normally available to AHSS researchers in general. By and large most research grants in AHSS subjects tend to range between £5–£250k in value and many last no longer than between 12–24 months, allowing little time and resources for the development of novel strategies to digital research dissemination. Similarly, not all research enquiries might lend themselves well to digitisation due to the ethical, epistemological and practical concerns referred to above. Questions of authorship, the suitability of metrics for assessing impact and dissemination ethics continue influencing debates on the merits of digital dissemination and shall remain points of contention in the foreseeable future. However, as has been demonstrated above, there are circumstances where employing digital dissemination practices, DOIs and ORCIDs numbers is highly appropriate and could potentially lead to raising the profile of research in AHSS domains, demonstrating that this same research is capable of generating its theory either independently or in true collaboration with science partners.

Whilst this paper has explored some of the (perceived) differences between AHSS and non-AHSS uses of digital approaches to data sharing and management, I would suggest that, based on preliminary discussions had, there are also many similarities between researchers and how they relate to their data, regardless of their disciplinary background. In future it may therefore be useful to explore the commonalities between disciplines alongside differences to help foster interdisciplinary approaches to research data management both practically and epistemologically, using a bottom-up approach.
