# Supplementary material for: Problematizing Digital Research Evaluation using DOIs in Practice-Based Arts, Humanities and Social Science Research
Source: F1000Res. 2015 Jul 7;4:193. [Version 1] doi: 10.12688/f1000research.6506.1 (PMC4513688; doi:10.12688/f1000research.6506.1)
Supplement: Peer review history

# Peer review: Referee response for version 1

**DOI:** 10.5256/f1000research.6982.r9377

**Published:** 2015-07-17

**Authors:** Ben Johnson

**Affiliations:** 1 Higher Education Funding Council for England (HEFCE), Avon, UK

This article gives an insightful and valuable overview of the challenges and opportunities of adopting new practices of digital scholarship within AHSS research processes. Building on the work of the Wilsdon review and earlier studies into practices within arts, humanities and social sciences research (e.g. the Crossick review), this provides a timely description of the difficulties we face in implementing broad solutions to tricky problems within a diverse research base.

The diversity of research is often name-checked by those looking at the whole system and seeking to improve the way it works, but perhaps not fully understood. The specific process-oriented examples and case studies revealed here provide important contextual information to inform the sensible and sensitive roll-out of modern research management tools and approaches – it may be desirable, from a management and assessment perspective, to see universal adoption of ORCIDs, DOIs and so on, but this isn’t as easy as it sounds, and this article helps to explain why this might be the case while providing helpful examples of where it has worked and suggestions for ways forward.

A particular problem is the complex and finely balanced nature of the relationship between different facets of the research process. While ethics, IP, copyright, digitisation, licensing, identification, citation, metrics and credit are often thought of as somewhat bounded issues that can be solved by ‘fixing the plumbing’ (e.g. by introducing ORCIDs), this article reminds us how complex their linkages are within the research process and how upsetting just one part of the balance can introduce vulnerabilities into the whole system.

The examples given here about data management within arts disciplines are rich and informative, and justify a bottom-up approach to managing this agenda (as called for in the conclusion). It is already clear that ‘data’ means different things to different disciplines; even across (largely STEM) disciplines that generate numerical data as a primary output, one finds large variations in definitions, standards, practices and expectations that tend to muddle us. Extending the meaning of ‘data’ to include all inputs and outputs that inform and support the insights generated from the research process is a laudable aim of those seeking to increase the transparency, robustness, replicability, dissemination and impact of research; doing so in a way that take sufficient account of the complex dependencies between anonymity, confidentiality, intellectual property, ethical propriety and so on is a particular challenge within AHSS research and one that is perhaps not given sufficient attention by those operating at the ‘macro’ level of research administration, assessment and policy development.

Beyond data, the particular problems of contributor anonymity, delineating roles within collaborations with non-academic colleagues, the invalidity of digital simulacra of real-world artistic artefacts, the complexity of documentation of data drawn from a wide range of often privately-owned sources… these are problems that are not felt by colleagues in STEM (the group of disciplines from which it is often felt that moves to ‘digitise’ research are flowing). The assignment of DOIs, ORCIDs, OA licences and so on to the outputs of research operating in this environment is tricky and fraught with real dangers that will require careful further investigation. There is a clear need to need to tease out the limitations of these new aspects of the research ‘plumbing’ within disciplines, explore novel solutions, find what works and what doesn’t, and seek a sensible way forward.

At the heart of this is the question of ethics. The close dependency between more open and transparent scholarly communication practices and more effective research integrity are not disputed, but this is often used to justify a conclusion that ‘open’ is ‘better’ in all cases. The examples above, particularly of ethnographic research, reveal that ethical limitations within disciplinary practice often inform models of communication in a way that might hinder openness, and that this is entirely appropriate in the disciplinary context. This at first appears to fly in the face of the very idea of “open science”, but in practice it only underlines the need for context-specific approaches to openness that take sufficient accounts of the ethical practices within disciplines. Clear delineation is needed, though, between genuine ethical considerations and those simply borne of more affected academic-cultural norms or resistant to practical change – we need to head off any unfair accusations of ‘special treatment’ being granted to these disciplines purely on political grounds. We need to better understand this problem, so that we can more effectively and sensitively tailor our approaches to achieve open research communication in a way that respects good research practice in all disciplines.

Finally, the question of metrics. Central to the arguments made above, and elsewhere, is a concern that the ‘plumbing’ of DOIs, ORCIDs, Web of Science coverage etc. is insufficient to enable the accurate capture of research outputs within AHSS, and therefore the metrics systems that depend on counting research outputs will unfairly discriminate against these disciplines. As the article states, “the upshot of this is that those disciplines that are less digitally oriented, are likely to obtain unhelpful metric ratings.” In my view, this masks a more pressing issue, which is that metrics are most applicable to those disciplines that ‘chunk’ their outputs into easily quantifiable forms, with quantifiable relationships to one another, with quantifiable citation practices, quantifiable(ish) contributions of academics to the research, and so on. It’s clear from the above, and from my own discussions with AHSS researchers, that the problems of quantifying AHSS research are not only related the coverage of DOIs and ORCIDs, and we should be careful not to assume that we entirely fix the issue of metrics by fixing the plumbing (even though we might get a few ‘quick wins’ in a few areas).

I have read this submission. I believe that I have an appropriate level of expertise to confirm that it is of an acceptable scientific standard.

# Peer review: Referee response for version 1

**DOI:** 10.5256/f1000research.6982.r9381

**Published:** 2015-07-13

**Authors:** Anne Galliot

**Affiliations:** 1 Centre for Research and Development (Arts), University of Brighton, Brighton, UK

This article explores the specific needs of arts, humanities and social sciences (AHSS) data management, whether these are adequately served by existing data management systems, and whether they are appropriately considered in future data management developments.

It highlights issues with the quantification and digitisation of information for research assessment and problematizes the concept of data itself, a notion that does not easily translate into AHSS disciplines. The author identifies this as a barrier to AHSS researchers engaging with the digital research information systems such as ORCID: it simply has little relevance to them.

Links are made between current practices in research data management, licencing, authorship and ethics, and the myriad considerations that might arise in AHSS projects for which we do not at present have satisfactory frameworks. The article reiterates the inadequacy of metrics to capture research impact, and indeed, excellence, in the AHSS; this is corroborated by their absence in the latest Research Excellence Framework under Panel D, as well as by the recent findings of the Independent Review of the Role of Metrics in Research Assessment and Management, which indicate that it is not currently feasible to assess research outputs or impacts in the REF using quantitative indicators alone ( Wilsdon *et al.*, 2015).

Finally, the author presents two research projects where some of the issues highlighted are being targeted, specifically by the creation of digital data sets and open access monograph and arts catalogues, in the formulation of research method and output. These projects are interdisciplinary and, crucially perhaps, ‘well-funded and usually include an element of non-AHSS research’. While it is clear that interdisciplinarity can contribute much to exploring these issues, it is uncertain whether tools and findings from these projects will fare better that the KULTUR project, which AHSS researchers are mainly unaware of.

This is an opinion article, based on the author’s experience of working, and interviews, with researcher at Goldsmith. It certainly reflects my experience as Research Adviser for a College of Arts and Humanities. It may have been useful to reference some of the arguments referred to in the introduction, although the paucity of literature on these very current issue may have played against this.

This article raises important questions about the definitions, ethical dimension, and the process of digitisation of research data, as well as about sector endeavours to quantify research impact and excellence. It works as a thought-provoking piece, begging many follow-on questions: How might we help AHSS researchers expand a definition of research data that will be relevant to them, and how do we enable the sector to acknowledge and redress the generalisation of its definition in favour of STEM disciplines? How might we ensure that the findings of KAPTUR and future projects are taken into consideration and their toolkits used? How might we address the apparent contradictions between copyright and intellectual property considerations and open access policies? How might we ethically define authorship in cases where research participants have contributing to shaping the data?

I hope this article leads to many more engaging with these questions in more depth.

I have read this submission. I believe that I have an appropriate level of expertise to confirm that it is of an acceptable scientific standard.

# Response

**Published:** 2015-07-14

**Authors:** Muriel Swijghuisen Reigersberg

**Affiliations:** Goldsmiths, University of Lonon, UK

Dear Anne,

Thank you for your review and supportive comments. I think based on these I ought to have better contextualized this submission, as it is somewhat unusual. This paper is in fact part of the conference proceedings of the CASRAI-ORCID conference, Barcelona, May 2015 on Research Evaluation, with an emphasis on emerging practice in the Humanities and Social Sciences http://www.orcid-casrai-2015.org/. The content of this paper was broadly discussed in a lively panel entitled: “Beyond Authorship: Recognising all research contributions."

As such this paper therefore, was given prior to the official launch of the Hefce Metrics review http://www.hefce.ac.uk/pubs/rereports/Year/2015/metrictide/. Hence materials of this review were not included in the first versions of this paper. However, now that the report has been launched I shall be able to include a link + DOI for it and draw on some of the literature it mentions, which, as you suggest, would be very useful indeed. I would recommend that anyone reading or reviewing my submission also reads the Hefce report to contextualise this paper.

Secondly, this submission is not actually an opinion piece. However, F1000 - being a predominantly bio-medical journal did not cater to arts, humanities and social science electronic 'templates' (if ever there were any), so the 'opinion' format was the only one suited for my particular submission. Thankfully editorial staff and conference organisers were very understanding about this small logistical hurdle and I am grateful to have been given the opportunity to contribute as part of the special theme on communicating science stream.

Hope that's useful.
